# Supplementary material for: Empirical methods for controlling false positives and estimating confidence in ChIP-Seq peaks
Source: BMC Bioinformatics. 2008 Dec 5;9:523. doi: 10.1186/1471-2105-9-523 (PMC2628906; doi:10.1186/1471-2105-9-523)
Supplement: Additional File 1 — A variety of html documents from the USeq web site detailing the available applications, their best usage, output file type descriptions, command line menus, etc. [file 1471-2105-9-523-S1.zip › USeqUserGuides/JavaDoc.zip!/JavaDoc/util/bio/wrappers/class-use/BL2SeqHit.html]

Uses of Class util.bio.wrappers.BL2SeqHit


|  |  |  |
| --- | --- | --- |
| **Overview** \| **Package** \| **Class** \| **Use** \| **Tree** \| **Deprecated** \| **Index** \| **Help** | |  |
| PREV   NEXT | **FRAMES**    **NO FRAMES**     **All Classes** |  |


---


## **Uses of Class util.bio.wrappers.BL2SeqHit**

| Packages that use BL2SeqHit | |
| --- | --- |
| **bioroot** |  |
| **util.bio.wrappers** | Wrappers for other bio applications. |

Uses of BL2SeqHit in bioroot

| Methods in bioroot with parameters of type BL2SeqHit | |
| --- | --- |
| `boolean` | `BlastMatcher.matches(BL2SeqHit[] hits)` |

Uses of BL2SeqHit in util.bio.wrappers

| Methods in util.bio.wrappers that return BL2SeqHit | |
| --- | --- |
| `BL2SeqHit[]` | `BL2Seq.blastFASTAFiles(java.io.File seq1File, java.io.File seq2File)`             Uses BLAST bl2seq program to align two sequences found in the seq files, won't work on windows? |
| `BL2SeqHit[]` | `BL2Seq.getHits()` |
| `BL2SeqHit[]` | `BL2Seq.parseBl2SeqResults(java.lang.String[] data)`             This takes an array of BLAST bl2seq results and parses the info into an array |

---


|  |  |  |
| --- | --- | --- |
| **Overview** \| **Package** \| **Class** \| **Use** \| **Tree** \| **Deprecated** \| **Index** \| **Help** | |  |
| PREV   NEXT | **FRAMES**    **NO FRAMES**     **All Classes** |  |


---
